# HIV/AIDS Related Stigma and Discrimination against PLWHA in Nigerian Population

**DOI:** 10.1371/journal.pone.0143749

**Published:** 2015-12-10

**Authors:** Maznah Dahlui, Nazar Azahar, Awang Bulgiba, Rafdzah Zaki, Oche Mansur Oche, Felix Oluyemi Adekunjo, Karuthan Chinna

**Affiliations:** 1 Department of Social and Preventive Medicine, Faculty of Medicine, University of Malaya, Kuala Lumpur, Malaysia; 2 Department of Medical Laboratory Technology, Faculty of Health Sciences, Universiti Teknologi MARA, Pulau Pinang Campus, Pulau Pinang, Malaysia; 3 Julius Centre University of Malaya, Department of Social and Preventive Medicine, Faculty of Medicine, University of Malaya, Kuala Lumpur, Malaysia; 4 Department of Community Health, Usmanu Danfodiyo University, Sokoto, Nigeria; 5 Department of Development Studies, Faculty of Economics and Administration, University of Malaya, Kuala Lumpur, Malaysia; University of Missouri-Kansas City, UNITED STATES

## Abstract

**Background:**

HIV/AIDS remain a major public health concern in Nigeria. People living with HIV/AIDS (PLWHA) face not only personal medical problems but also social problems associated with the disease such as stigma and discriminatory attitudes. This study provides an insight into HIV/AIDS related stigma and discrimination against PLWHA in Nigeria.

**Methods:**

The data for this study was extracted from the 2013 Nigeria Demographic and Health Survey conducted by the National Population Commission. All men and women aged 15–49 years, permanent residents and visitors of the households were eligible for the interview. Several questionnaires were used in the survey, some covering questions on HIV/AIDS.

**Results:**

A total of 56 307 men and women aged 15–49 years participated in this national survey. About half of the population in Nigeria have HIV stigma. Younger persons, men, those without formal education and those within poor wealth index are more likely to have stigma towards PLWHA. In addition, married people are more likely to have stigma on PLWHA and are more likely to blame PLWHA for bringing the disease to the community. Also about half of the population discriminates against PLWHA. However, those with higher levels of education and those from higher wealth index seem to be more compassionate towards PLWHA. About 70% in the population are willing to care for relative with AIDS, even more so among those with higher level of education.

**Conclusion:**

There is a high level of HIV stigma and discrimination against PLWHA in the Nigerian population. Education seems to play a major role in the society with respect to HIV stigma and discrimination against PLWHA. Educating the population with factual information on HIV/AIDS is needed to reduce stigma and discrimination towards PLWHA in the community.

## Introduction

Human Immunodeficiency Virus (HIV) infection and Acquired Immune Deficiency Syndrome (AIDS) remain a major public health problem in Nigeria which is only second behind South Africa in terms of the number of people with HIV/AIDS [1]. Despite of numerous efforts on prevention and treatment of HIV/AIDS, this infection is still an epidemic and affects healthy people as well. One of the most significant challenges for the success in controlling HIV/AIDS infection is stigma and discrimination. Existences of prejudice and discrimination against people with specific diseases have been well established [2–4]. Stigma and discrimination tend to isolate PLWHA from the community and give negative impact on their quality of life [5–7]. Even though the prognosis of PLWHA could be improved with anti-retroviral treatment, they still have to face condemnation and isolation from colleagues, family and community because people around them are conscious about their HIV status [8]. On the other hand, when PLWHA are shown compassion by the community, they are likely to take protective precautions in their sexual behavior [6] and be more open about their HIV status [5]. This problem of stigmatization and discrimination among PLWHA is particularly more widespread in sub-Saharan Africa including Nigeria, due to the weak health system coupled with poor legal and ethical framework [9].

Stigmatization can lead to prejudicial actions and thoughts among governments, communities, health care providers, employers, family members and colleagues [10]. There could be a number of health problems among HIV/AIDS patients, such as: loneliness, isolation, low self-esteem, identity crises and lack of interest towards prevention of HIV/AIDS [4]. These patients also lack motivation in practicing preventive measures [11], their care seeking behavior is low [12], and do not participate in routine HIV testing [13]. Many pregnant women avoid voluntary counseling and testing [14–16]. Perceived HIV stigma has an indirect effect on people’s quality of life [17]. In a systematic review conducted by Bharat (2011) [18], approximately one third to half of the respondents including the health care providers had blamed PLWHA for bringing the disease into the community. In terms of their role in the community development, PLWHA were assumed as not being able to contribute to their society [5]. In most situations, in order to prevent social rejection, PLWHA will not disclose their HIV status to avoid being isolated from participating in the socio-cultural events [18–20]. Stigma will augment the prevalence of HIV/AIDS by halting the delivery of effective social and medical support because PLWHA are not able to interact with their families and communities which is supposed to make them feel complete, secured and be a part of the society [21].

HIV infected patients might also experience a number of undesirable conditions in their life such as: hostility, denial of gainful employment [22], forced resignation or forced early retirement, delivery of poor quality treatment and segregation in hospital wards [23]. Discrimination and exclusion from the community are the consequences of stigma that are usually experienced by PLWHA. As a result, HIV-infected patients prefer not to disclose their HIV sero-status and continue to engage in high-risk behavior [24–25].

Issues pertaining to availability, affordability and accessibility of treatment and care of PLWHA have been cited for the poor control of HIV infection. However, how HIV stigma affects PLWHA are not well understood. Previous studies have revealed that eliminating stigma in the population will increase the acceptance of an individual as well as community towards PLWHA [26]. Understanding and addressing the barriers of stigma and discrimination are the important factors that need to be explored. In line with these, our study was aimed to assess the HIV stigma and discriminatory practices among women and men aged 15–49 years in Nigeria.

## Materials and Methods

The data for this study were taken from the 2013 Nigeria Demographic and Health Survey (2013 NDHS), conducted by the National Population Commission. In this survey, women and men aged 15–49 years of age, permanent residents or visitors of the households were included. A stratified three-stage cluster design was used in this study. Prior to the commencement of the study, a complete listing of the household was obtained. Training on how to use the Global Positioning System (GPS) receiver was delivered to the enumerators [27].

In the survey, besides others, there were questionnaires on HIV/AIDS, one to be answered by the men and the other to be answered by the women. The questionnaires were administered in different languages namely Igbo, Yoruba and Hausa in order to overcome language barriers that may confound the results. In the questionnaire there were sections on stigma and discrimination towards PLWHA, acceptance on various issues pertaining to stigma and discrimination such as willingness to take care of their infected family members, preference to buy vegetables from HIV-infected vendor, discrimination towards female infected teacher, perception on whether HIV positive people should be ashamed of themselves and perception on whether HIV infected people should be blamed for bringing the disease to the community. Socio-demographic information such as age, highest educational attainment, marital status and household monthly income was also gathered.

Inclusion criteria for this study were women and men aged 15–49 years of age in the household and those who gave consent to participate in the study. Participation was solely voluntary, since the information gathered were concerning personal and sensitive issues such as their sexual relationship, stigma and attitudes of discrimination towards PLWHA. The interviewers ensured that participants had been informed about the study protocol, upon which a written informed consent was obtained. Ethical clearance to conduct NDHS was obtained from the National Health Research Ethics Committee of Nigeria, Federal Ministry of Health, Abuja, Nigeria. NDHS data are public access data and were made available to us upon request by Measure DHS.

### Analysis

Statistical Package for the Social Sciences (SPSS) version 20 with complex samples procedure was used for the analysis. Frequency and percentages were used to describe the demographic characteristics of the respondents. The univariate logistic regression analysis procedure in the complex samples add-on module in SPSS was used to test the differences between the male and female subjects on HIV stigma discrimination against PLWHA. The multivariate logistic regression procedure was used to test for factors associated with HIV stigma and discrimination against PLWHA.

## Results

A total of 56 307 men and women aged 15–49 years had participated in the study. Majority of the participants were aged 21–30 years (34.6%), had secondary education (40.5%), were able to read (62.1%), in the richer wealth index (22.2%), resided in rural areas (59.7%), resided in North West region (24.5%), Christians (51.4%) and were married (61.6%). The socio-demographic characteristics by gender are shown in Table 1.

**Table 1:** Socio-demographic characteristics of participants.

Socio-demographic characteristics	n (%)		
	Women	Men	Total
	(n = 38 948)	(n = 17 359)	(n = 56 307)
Age (years)			
- 11–20	10 186(26.2)	4 667(26.9)	14 853(26.4)
- 21–30	13 820(35.5)	5 651(32.6)	19 471(34.6)
- 31–40	9 304(23.9)	4 311(24.8)	13 615(24.2)
- 41–50	5 638(14.5)	2 730(15.7)	8 368(14.9)
Highest Educational Attainment			
- No formal education	13 740(35.3)	3 354(19.3)	17 094(30.4)
- Primary education	7 104(18.2)	2 979(17.2)	10 083(17.9)
- Secondary education	14 407(37.0)	8 390(48.3)	22 797(40.5)
- Higher education	3 697(9.5)	2 636(15.2)	6 333(11.2)
Literacy levels			
- Illiterate	17 208(44.4)	4 049(23.4)	21 257(37.9)
- Able to read	21 552(55.6)	13 218(76.6)	34 770(62.1)
Wealth index			
- Poorest	6 602(17.0)	2 646(15.2)	9 248(16.4)
- Poorer	7 515(19.3)	3 033(17.5)	10 548(18.7)
- Middle	8 001(20.5)	3 538(20.4)	11 539(20.5)
- Richer	8 450(21.7)	4 042(23.3)	12 492(22.2)
- Richest	8 380(21.5)	4 100(23.6)	12 480(22.2)
Locality			
- Urban	15 545(39.9)	7 144(41.2)	22 689(40.3)
- Rural	23 403(60.1)	10 215(58.8)	33 618(59.7)
Geographical zone			
- North Central	6 251(16.0)	3 018(17.4)	9 269(16.5)
- North East	6 630(17.0)	2 843(16.4)	9 473(16.8)
- North West	9 673(24.8)	4 131(23.8)	13 804(24.5)
- South East	4 462(11.5)	1 681(9.7)	6 143(10.9)
- South South	6 058(15.6)	3 035(17.5)	9 093(16.1)
- South West	5 874(15.1)	2 651(15.3)	8 525(15.1)
Religion			
- Christian	19 838(50.9)	8 974(51.9)	28 812(51.4)
- Islam	18 578(47.7)	8 134(47.1)	26 712(47.6)
Marital Status			
- Married	26 403(67.8)	8 292(47.8)	34 695(61.6)
- Unmarried	12 545(32.2)	9 067(52.2)	21 612(38.4)

**Fig 1:**
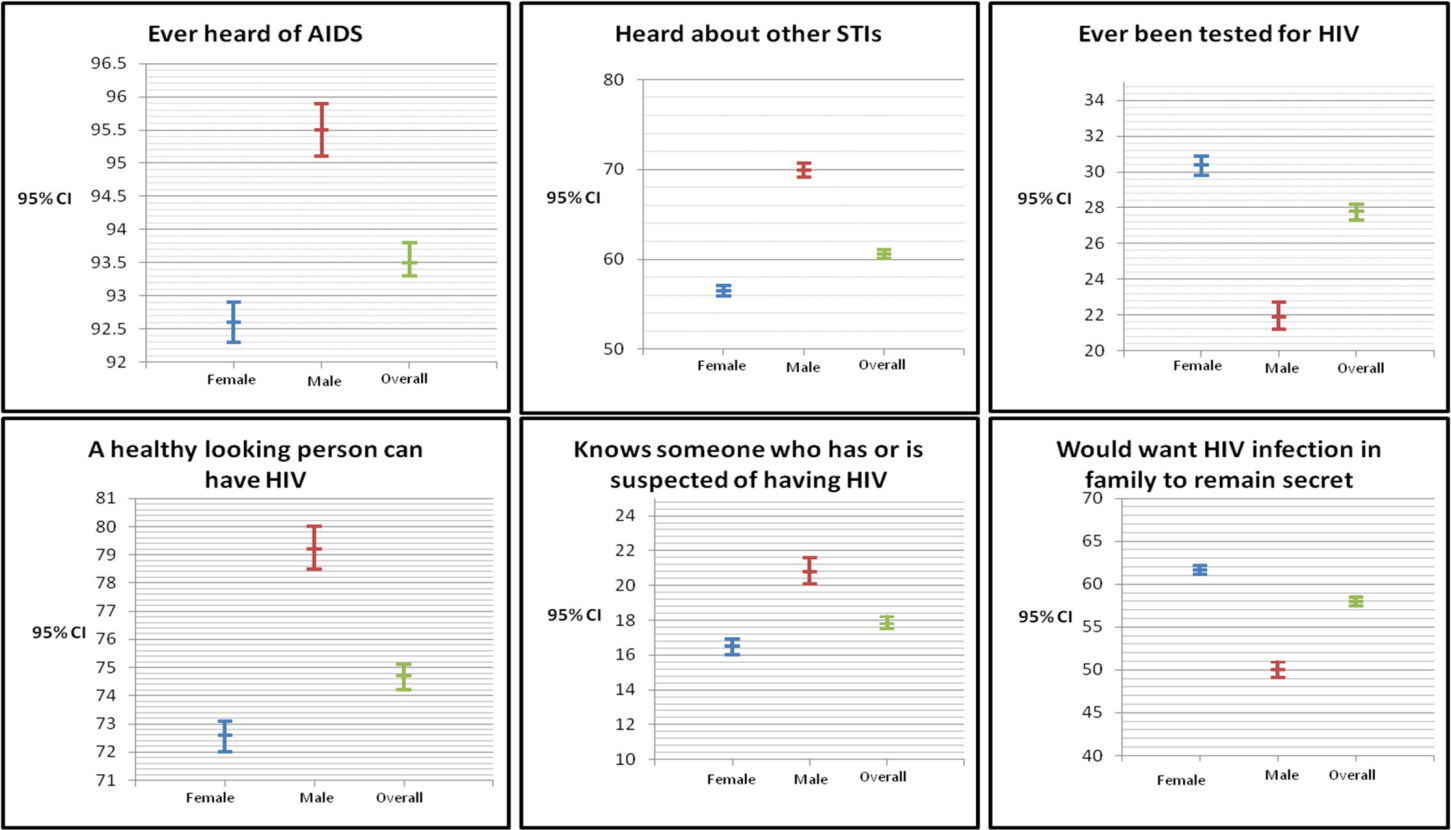
HIV/AIDS related knowledge and behaviour.

Results for HIV/AIDS related knowledge and behavior, overall and by gender, are presented in Fig 1. Overall, over 90 percent have heard of AIDS. With regards to STI, more males (70%, CI 69, 71) have heard about it compared to the females (57%, CI 56, 57). Relatively, more females (30%, CI 30, 31) have been tested for HIV compared to the males (22%, CI 21, 23). Overall, more than three quarters of the study subjects agreed that a healthy looking person may have HIV. Relatively, more males (21%, CI 20, 22) knew someone who has, or is suspected of having HIV compared to the females (17%, CI 16, 17). More women (62%, CI 61, 62) would want HIV infection in family to remain secret compared to 50% (CI 49, 51) among the males.

**Fig 2:**
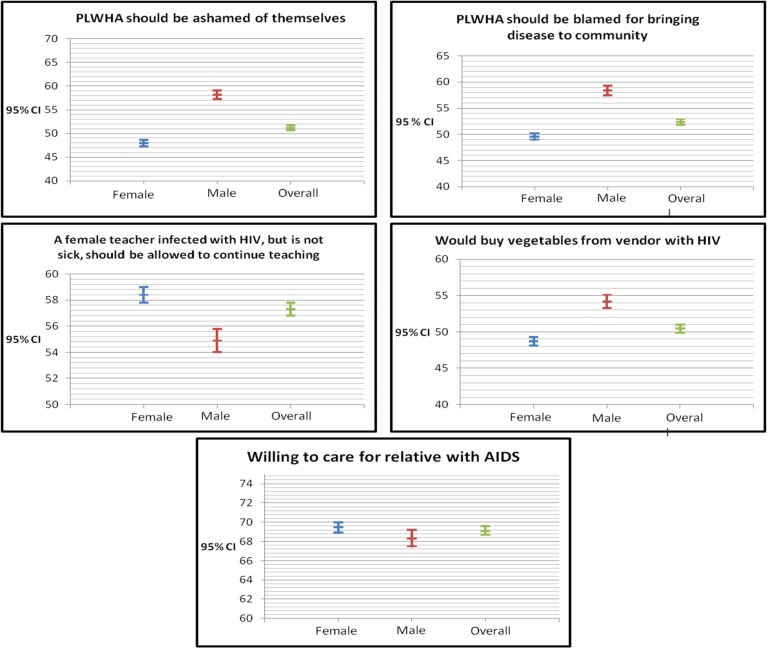
Stigma and discrimination against PLWHA.

Results from analyses on stigma and discrimination against PLWHA, overall and by gender, are presented in Fig 2. In this study HIV stigma was assessed using two questions; that people with HIV should be ashamed of themselves and that people with HIV should be blamed for bringing the disease into the community. Discrimination was assessed through three questions; whether female teachers infected with HIV, but is not sick, should be allowed to continue teaching, willingness to care for a relative with AIDS and willingness to buy vegetables from a vendor with HIV. Overall, about 50% of the population were in agreement that PLWHA should be ashamed of themselves and should be blamed for bringing the disease to the community, the level of agreement being slightly more among the males compared to the females (about 60% vs 50%). About 60% agreed that a female teacher infected with HIV, but is not sick, should be allowed to continue teaching and about 50% would buy vegetables from vendor with HIV infection. Overall, about 70% in the population are willing to care for relatives with AIDS.

Table 2 shows the results from multivariate analysis on factors associated with HIV stigma. Younger persons, males, those with lesser education and those in the lower wealth index tend to agree more that people with HIV should be ashamed of themselves and that people with HIV should be blamed for bringing the disease into the community.

**Table 2:** Socio-demographic factors associated with perceived stigma towards PLWHA.

Socio-demographic characteristics	HIV Stigma	
	People with HIV should be ashamed of themselves	People with HIV should be blamed for bringing disease to community
	Adjusted OR (95% CI)	Adjusted OR (95% CI)
Age (years)		
- 11–30	1.20(1.14–1.26)	1.31(1.25–1.37)
- 31–50	1.00	1.00
Sex		
- Men	1.76(1.68–1.84)	1.75(1.67–1.83)
- Women	1.00	1.00
Highest Educational Attainment		
- No formal education	1.75(1.58–1.94)	2.44(2.19–2.71)
- Primary education	1.39(1.29–1.50)	1.51(1.41–1.63)
- Secondary and higher	1.00	1.00
Literacy levels		
- Illiterate	1.08(0.99–1.19)	0.96(0.87–1.05)
- Able to read	1.00	1.00
Wealth index		
- Poor	1.92(1.80–2.05)	2.14(2.00–2.29)
- Middle	1.32(1.24–1.40)	1.42(1.34–1.51)
- Rich	1.00	1.00
Locality		
- Urban	1.10(1.05–1.16)	1.14(1.09–1.20)
- Rural	1.00	1.00
Marital status		
- Married	0.98(0.93–1.03)	1.06(1.00–1.11)
- Unmarried	1.00	1.00

Results from multivariate analysis on factors associated with HIV discrimination are presented in Table 3. Women, those with higher level of education and those in the middle and rich wealth index category tend to agree more that a female teacher infected with HIV, but is not sick, should be allowed to continue teaching. Those with higher level of education are more willing to care for a relative with AIDS. Men, those with higher level of education, those who are able to read, those in the rich wealth index category and those staying in urban locations tend to agree more that they would buy vegetables from a vendor with HIV.

**Table 3:** Factors associated with acceptance towards PLWHA.

Socio-demographic characteristics	HIV Discrimination		
	A female teacher infected with HIV, but is not sick, should be allowed to continue teaching	Willing to care for relative with AIDS	Would buy vegetables from vendor with HIV
	Adjusted OR (95% CI)	Adjusted OR (95% CI)	Adjusted OR (95% CI)
Age (years)			
- 11–30	1.00	1.00	1.00
- 31–50	1.10(1.05–1.15)	1.08(1.02–1.13)	1.08(1.03–1.13)
Sex			
- Men	1.00	1.00	1.00
- Women	1.25(1.20–1.31)	1.10(1.05–1.16)	0.86(0.82–0.90)
Highest Educational Attainment			
- Primary	1.00	1.00	1.00
- No formal education	1.04(0.97–1.13)	1.01(0.93–1.09)	1.48(1.36–1.60)
- Secondary and higher	1.57(1.46–1.69)	1.27(1.17–1.37)	1.38(1.28–1.48)
Literacy levels			
- Illiterate	1.00	1.00	1.00
- Able to read	1.06(0.97–1.15)	1.03(0.94–1.13)	1.53(1.40–1.67)
Wealth index			
- Poor	1.00	1.00	1.00
- Middle	1.27(1.19–1.35)	1.18(1.11–1.26)	1.20(1.12–1.28)
- Rich	1.37(1.29–1.46)	1.05(0.98–1.13)	1.30(1.22–1.39)
Locality			
- Rural	1.00	1.00	1.00
- Urban	1.09(1.03–1.14)	0.98(0.93–1.03)	1.27(1.21–1.33)
Marital status			
- Unmarried	1.00	1.00	1.00
- Married	1.06(1.01–1.12)	1.04(0.98–1.10)	1.14(1.08–1.20)

## Discussion

Negative perceptions towards PLWHA are some of the common manifestations of AIDS stigma which leads to discrimination and prejudice attitudes. Consistent with previous studies [4,17,25,28], our findings showed that there is a high level of stigma and discrimination towards PLWHA in the Nigeria population. Stigma and discrimination could have significant adverse effects on the daily lives of PLWHA. This issue tends to create a hidden epidemic of the disease based on fear, misinformation, socially-shared ignorance and denial [4,29]. In a study conducted by Alubo et al. (2002) [30], aimed at determining the levels of perception, knowledge and attitude towards PLWHA in the North-central Nigeria, it was found that the level of acceptance was low and the level of rejection was high towards PLWHA by the community members. Findings from the 2002 National HIV and AIDS Household Survey revealed that 80.8% of participants refused to sleep together in the same room with someone who has HIV infection and 94.5% of respondents would not even have conversation with HIV-infected person [31]. Stigma and discriminatory attitudes towards HIV/AIDS lead to secrecy and denial among PLWHA which form catalysts for HIV transmission [32].

In our study, among the factors tested, age, gender, education and wealth index were significantly associated with HIV stigma. Younger persons, males, those with lesser education and those in the lower wealth index tend to agree more that people with HIV should be ashamed of themselves and that people with HIV should be blamed for bringing the disease into the community. These findings are similar to that of several other studies. It is interesting to note that those with higher levels of education and in the higher wealth index are more sympathetic towards PLWHA. This could be due to higher awareness among the educated and wealthy ones on the prognosis of PLWHA and the availability of anti-retroviral treatments.

The HIV status of a person puts forward negative impact on their access to health and education services as well as discrimination in their working life. Based on this study, nearly half (40%) of people in the population do not agree that female teachers infected with HIV should be allowed to continue teaching, even when they are not sick. In developing countries teachers are highly respected and looked upon in the communities as role models and they are seen as those responsible in teaching moral values among the children. When infected with HIV, these teachers, especially the females, seem to lose the respect in the community. However, women, with higher level of education and those in the middle and rich wealth index category are more tolerant in this regard. Our finding corroborates with Oyediran et al. (2005) [33] who found that about two-third the Nigerian population agreed that office colleagues who have been infected with HIV/AIDS should not be allowed to continue working. In another study on unemployment, half of the respondents who had lost their jobs in the preceding 12 months reported that it was due to their HIV sero-status [34]. Most of the employees with HIV/AIDS suffer prejudice attitudes in their workplace from supervisors and colleagues in the form of social isolation, ridicule and discriminatory practice [35–36].

There is increasing concern about family caregivers’ reluctance to care for and treat family members with HIV/AIDS. As a result of this, most PLWHA would not disclose their HIV status even to their family members to avoid distancing reactions and discriminatory practices towards them. Studies have found that family caregivers also possess stigma and prejudice attitudes towards their own family members who have HIV/AIDS [32]. Based on this study, about 70% in the Nigerian population are willing to care for a relative with AIDS which goes to show the level of empathy showed by Nigerians towards PLWHA. A study conducted among the nursing students in New Delhi reported that more than half of the respondents were willing to take care of PLWHA if they are provided with relevant training and guideline [37]. Our findings also showed that those with higher levels of educational are more willing to take care for their relatives with AIDS. Thus, providing sufficient guidelines and education related to the disease prognosis, we can expect even more Nigerians to resolve this discrimination and provide the necessary care for their relative with AIDS.

In this survey, question on willingness to buy vegetables from HIV-infected vendor was used to assess how people associate themselves with PLWHA outside their domains. Our results showed that only half of the population indicated willingness to buy vegetables from HIV-infected vendor, though men, those with higher level of education, those who are able to read, those in the rich wealth index category and those staying in urban locations tend to be more tolerant in this aspect. This is a very serious level of discrimination towards PLWHA. In order to have normal lives within the community, PLWHA may adopt certain coping mechanisms like concealing their HIV status since people refuse to trade with them [38].

The findings from this study call for the need to promote positive and acceptable attitudes towards PLWHA in the Nigerian population. Programs should be put in place to increase the awareness on HIV/AIDS in the population, promote compassion towards PLWHA and emphasize on respect for the rights of PLWHA. A study conducted in South Africa, identified four areas that need to be addressed to combat HIV stigma; fostering awareness and knowledge among the public, educating PLWHA, advocating for the rights of infected persons and providing emotional as well as physical support [34]. There is ample evidence that eliminating stigma and discrimination will result in higher acceptance of PLWHA by family members and communities [39]. It is therefore important to implement strategies and programs to eradicate discriminatory attitudes and practices towards PLWHA.

Our study had determined the magnitude of HIV stigma and discrimination against PLWHA in Nigeria. More epidemiological studies need to be conducted to understand the impact of stigma and discrimination on prevention of HIV/AIDS. Future studies should also focus more on the HIV/AIDS-education or intervention programs that aim to increase the knowledge and awareness of population in the communities, especially among the rural communities. By increasing the populations’ knowledge on HIV/AIDS, stigma and discrimination towards PLWHA could be reduced.

## Conclusion

This study observed high discriminatory attitudes and practices towards PLWHA; however about 70% of the population are willing to care for relatives with HIV/AIDS. In order to combat HIV/AIDS epidemic in Nigeria, issues pertaining to stigma and discrimination need to be addressed. Health promotion campaigns should incorporate a shift from fear to care for PLWHA as this is important for effective preventive measures. As stigma and discrimination continues to be a hidden factor that acts as impediment for the effective prevention program, policy makers need to strengthen the HIV/AIDS intervention and health education program in local communities in Nigeria. Educating the population with respect to improve their understanding on HIV/AIDS transmission and control are crucial to reduce this menace in Nigeria. Education and knowledge are believed to be the vanguard for the disease prevention. Behavioral change strategies should be delivered among the population in order to impede the spread of the disease.
